# Wild chimpanzees modify modality of gestures according to the strength of social bonds and personal network size

**DOI:** 10.1038/srep33864

**Published:** 2016-09-21

**Authors:** Anna Ilona Roberts, Sam George Bradley Roberts

**Affiliations:** 1 Department of Psychology, University of Chester, Chester; Parkgate Road, Chester CH1 4BJ, UK

## Abstract

Primates form strong and enduring social bonds with others and these bonds have important fitness consequences. However, how different types of communication are associated with different types of social bonds is poorly understood. Wild chimpanzees have a large repertoire of gestures, from visual gestures to tactile and auditory gestures. We used social network analysis to examine the association between proximity bonds (time spent in close proximity) and rates of gestural communication in pairs of chimpanzees when the intended recipient was within 10 m of the signaller. Pairs of chimpanzees with strong proximity bonds had higher rates of visual gestures, but lower rates of auditory long-range and tactile gestures. However, individual chimpanzees that had a larger number of proximity bonds had higher rates of auditory and tactile gestures and lower rates of visual gestures. These results suggest that visual gestures may be an efficient way to communicate with a small number of regular interaction partners, but that tactile and auditory gestures may be more effective at communicating with larger numbers of weaker bonds. Increasing flexibility of communication may have played an important role in managing differentiated social relationships in groups of increasing size and complexity in both primate and human evolution.

Strong and enduring social bonds in primates are closely linked with fitness outcomes^1^, but the need to track and maintain these social relationships results in primate sociality being constrained by both temporal and cognitive factors^2^. These constraints give rise to primate groups with a multilevel structure containing hierarchically nested social levels, with individuals having a differentiated set of stronger and weaker social relationships with conspecifics^3^,^4^. Gestural communication–voluntary movements of the arms, head, body postures and locomotory gaits^5^,^6^,^7^–has been hypothesized to regulate social bonding in some multilevel societies^8^, because it provides an important channel through which individuals influence the behaviour and emotions of their social partners^5^. Gestures vary in modality from less intense visual gestures to more intense auditory long-range or tactile gestures^5^,^6^. Different types of social bonds may be associated with different patterns of gestural communication through the intensity of emotional arousal, whereby individuals express their own emotional arousal and also evaluate and process emotional arousal in others, in order to respond in an adaptive manner^9^,^10^,^11^,^12^,^13^,^14^,^15^,^16^,^17^.

Emotional arousal has been operationally defined as a state of physiological activation experienced as a change in heart rate^14^,^18^, cortisol secretion^19^,^20^,^21^,^22^,^23^ or nasal temperature^24^. Arousal change is associated with corresponding behavioural patterns, precipitated by exposure to different types of bonds with the social partners^25^,^26^,^27^,^28^. For instance, when a primate interacts with a conspecific with whom they have a history of prior aggressive interactions, this is associated with an increase in heart rate, indicating an aroused response^14^,^18^. In contrast, when a primate interacts with a conspecific with whom they have a positive social relationship, there is no increase in heart rate from baseline, indicating a lack of arousal^14^,^18^. Various indicators of arousal relating to communication have also been examined. It has been proposed that indices of arousal and arousal change can be classified along the dimension of communication intensity or strength such as the potency of its presentation (loudness), frequency and duration^25^,^29^. For instance, non-verbal loudness (rated on the scale from loud to soft) is correlated with self-reported measures of emotional arousal in humans^25^. Louder human voices are associated with higher arousal than quieter voices, providing further evidence that communication loudness can be used to indicate the intensity of arousal^30^,^31^,^32^. Moreover, other research in humans has revealed that the communication in itself can alter the arousal of the recipient^33^,^34^,^35^,^36^,^37^. It has been shown that communication associated with different levels of emotional arousal is associated with different behavioural, physiological and fitness outcomes in the recipients. High-arousal signals trigger a range of neurological and hormonal responses associated with increased heart rate and cortisol release^13^,^24^,^38^, which can influence the recipient’s health and survival^39^. High-arousal signals in primates such as alarm calls, calls produced by subordinates when threatened by dominants or calls by youngsters soliciting attention from their mothers can trigger automatic nervous system responses in recipients due to the acoustic properties of the vocalisations^40^,^41^,^42^,^43^. These ‘squeaks, shrieks and screams’ can be effective in changing affect and behaviour in recipients as with repetition they become highly arousing and are difficult to habituate to^40^,^41^. In contrast, in human mother-infant pairs and dog owners, low-arousal signals are associated with oxytocin release^44^, which has a stress buffering effect^45^ and is associated with improved immune response^46^. Oxytocin plays a key role in social bonding in humans^47^, and has experimentally been shown to be associated with affiliative behavior and proximity maintenance in dogs^48^. Oxytocin is also released in response to grooming^49^ and food sharing^50^ in wild chimpanzees.

Although high arousal states can be beneficial (e.g. in contexts such as predator defence), the stress caused by these states is generally maladaptive to both signallers and recipients. The intensity of the arousal state in communication can influence the effectiveness of behavioural coordination by influencing the efficiency by which recipients can infer temporal changes in the arousal states of the signallers. Responding appropriately to high-intensity communication is a relatively straightforward task because as arousal increases, the production by the signaller of the most common communication type associated with that emotion generally increases. In contrast, responding to low intensity signals is more difficult because as arousal declines, a greater variety of communication types are used^26^,^27^,^28^,^35^,^51^. Thus the emotional content of high-arousal signals (e.g. a dominance display by a male chimpanzee) tends to be unambiguous and may trigger a set of neural and physiological reactions in the recipient that leads to an appropriate response from the set of potential action opportunities^52^. In contrast, low arousal signals often contain less specific emotional information (e.g. anger displayed in a visual signal). It has been hypothesized that to respond appropriately to low arousal signals, the recipient has to determine whether or not the signal is directed at them and infer the signaller’s goals from contextual information relating to the identity of signaller, the relationship between the signaller and the recipient and the context (e.g. mating, aggression, travel)^53^,^54^. Low-arousal signals may thus require that the recipients integrate a wider range of sources of information in order to respond in an efficient manner, as compared to high-arousal signals^54^.

The need to consider contextual information relating to social relationships when responding to low intensity signals may impose time and cognitive demands on behavioural coordination because the number of dyads and triads of social relationships that have to be tracked and socially managed increases as a power function of the number of individuals in a group^55^. The relationship between social complexity (i.e. managing a more complex network of relationships) and neocortex size has previously been shown in relation to various aspects of primate sociality^55^. Primates with larger neocortices have higher rates of social play^56^, more complex male mating strategies^57^, higher levels of tactical deception^58^, are more likely to form coalitions^59^ and have a higher frequency of social learning^60^. Whilst this suggests that primates with larger neocortices do display a higher level of ‘social complexity’ in their behaviour, what is lacking is a systematic and detailed understanding of how contextual understanding of low emotional arousal as expressed in gestural communication is related to the nature of the bonded network–the number and strength of social bonds maintained with others.

Social network analysis offers a novel way to examine the role of communication in maintaining social bonds in primates^61^,^62^,^63^. In contrast with earlier analysis of social behaviour, social network analysis measures behaviours between dyads of individuals, and uses these to look at the differentiation and strength of ties across all individuals^61^. Thus, social network analysis can examine the structure of the group as a whole, in terms of different social behaviours and communication patterns^61^. In social networks analysis, each node usually represents an individual, and each edge (or ‘tie’) represents some measured social interaction (e.g. time spent in close proximity, rate of gesturing). The social network approach is grounded in the notion that the patterning of ties in which individuals are embedded has important consequences for these individuals. Central individuals maintain close proximity with numerous conspecifics and thus may serve as a centre for communication exchange. In contrast, peripheral individuals maintain rare or no close proximity with others and thus are located spatially at the margins of the social network.

In particular, the fission-fusion nature of chimpanzee society, where the association patterns change on a daily basis by means of the fission and fusion of subunits (known as parties or sub-groups)^64^ provides distinct challenges in managing a large and diverse set of social relationships^65^. A lack of predictability in conspecifics’ behavior is a major cause of stress^66^ and a fission-fusion social system increases this unpredictability, as the reproductive or dominance status of conspecifics may have changed after a period of absence. Chimpanzees only see some conspecifics infrequently, yet need to recognize, retain information and maintain long-standing relationships with all members of the community^65^. Chimpanzees thus maintain a variety of different types of social relationships with others, and the strength of social relationships in chimpanzees and other primates have been measured by different criteria, including duration of time spent feeding, travelling, resting, visually attending and grooming with the bonding partner^67^,^68^,^69^,^70^,^71^,^72^,^73^,^74^,^75^,^76^,^77^,^78^,^79^. Here we compare these measures with our measure of close proximity (duration of time spent within 10 m, per hour spent in the same party) to validate its use as an indicator of close social bonding. We hypothesize that the strength of chimpanzee social bonds (expressed as duration of time spent in close proximity) is not randomly distributed across group members but is associated both with modality of gestural communication and biological factors (reproductive status, age and sex similarity, kinship). Thus, we predict that strong proximity bonds will be associated with visual gestures and weak proximity bonds will be associated with auditory and tactile gestures, as chimpanzees use different modalities of gestures to maintain the different types of bonds. As individuals living in social groups have to both meet their own needs and coordinate their behaviour with others, we predict that this relationship will be seen across different types of contexts, including affiliative and antagonistic contexts^29^.

Moreover, individual chimpanzees display a large amount of variation in the number of conspecifics they maintain close proximity with. When chimpanzees have close proximity with only a small number of conspecifics, they may be able to maintain bonds with all network members based on visual gestures. However, when chimpanzees maintain close proximity to a larger number of conspecifics, they face higher time and cognitive demands on managing these more numerous social relationships. Therefore in larger proximity networks, there is an increasing number of the individuals with whom the proximity bonds are weaker and an increasing dissociation between the networks based on visual and tactile or auditory gestures. As these gestures vary in the time and cognitive demands on their perception, we predict that smaller proximity networks will be associated with the use of visual gestures, whereas larger proximity networks will be associated with more use of tactile and auditory gestures.

## Results

### Sampling effort

In this study, an average of 12.52 (range 8.33–18.63) hours of independent focal data per individual subject (N = 12 focal chimpanzees) was examined. Table 1 provides definitions of modalities of gestures, including gesture types, signaller and recipient distance, rates of production and normalized degrees (percentage of all connections chimpanzees had with others) across the 132 chimpanzee dyads. There was no statistically significant relationship between the total duration of observation and each of the gesture modality networks: auditory short-range (*C* = 1.085, *p* = 0.379), auditory long-range (*C* = 1.378, *p* = 0.191), visual (*C* = 0.910, *p* = 0.412) and tactile (*C* = 1.217, *p* = 0.317). This indicates sufficient sampling duration, in that the duration of observation for each chimpanzee dyad was not statistically related to the rate of gesturing between that dyad across each of the four gesture modalities.

### Gesture function and modality

First, we examined the relationship between the modality of gestures and their function. This analysis compared how the rate of gesturing in a particular modality (e.g. visual) was associated with gestures given in a particular context, including both affiliative (e.g. greeting) and agonistic contexts (e.g. threat to dominate). Table 2 gives definitions of all gesture functions and contexts. In this study, in all instances of gestural communication, the intended recipient of the communication was within 10 m of the signaller. In all models described in this section we controlled for sex, age, kinship and reproductive similarity. The details of all models are shown in Supplementary Tables S3–13 and the significant predictors for gesture function and modality are provided in Table 3. The rate of auditory long-range gestures was positively significantly associated with gestures produced in following contexts: threat to dominate, other threat, travel, copulation and pant-hoots. For auditory short-range gestures, the significant contexts were threat to dominate, copulation, greeting and gestures to give groom. Tactile gestures were significantly associated with reassurance, greeting, gestures to give groom and play. Finally, visual gestures were significantly associated with the following contexts: threat to dominate, food sharing, other threat, travel, copulation, greeting, gestures to mutually groom, gesture to receive groom, play, and pant-hoot.

### Proximity networks, visual attention, grooming, feeding, resting and travel

Second, we examined the predictors of the proximity bonds between dyads of chimpanzees. The chimpanzees maintained at least some proximity with 95.5% (range 82–100%) of all other focal individuals, but only maintained strong proximity bonds with a small number of these individuals. Three different binary networks of close proximity bonds were created, to identify bonds of different types. The ‘preferred, reciprocated close proximity bonds’ were defined as dyads that had reciprocated values of proximity association equal or above the mean plus half SD (i.e. both A to B and B to A had values of close proximity equal to or above 30.3 minutes duration per hour spent in the same party)^71^. 15.1% of all potential connections chimpanzees had with others (range 0–46%) were preferred, reciprocated close proximity bonds. Moreover, ‘preferred, non-reciprocated close proximity bonds’ were identified as dyads where the values of proximity association were equal or greater than the mean plus half SD, but the proximity was non-reciprocated (i.e. only A to B but not B to A had a duration of proximity equal or above the 30.3 minutes). Chimpanzees had preferred non-reciprocated close proximity bonds with 37.9% of potential proximity connections (range 18–55%). Finally, dyads of individuals who had values of proximity association equal or below the mean minus half SD (who spent 16.23 or less minutes in close proximity to each other per hour spent in same party) were defined as ‘non-preferred close proximity bonds’. In this network, 53.01% of all potential close proximity connections were non-preferred close proximity bonds (range 9–82%). In all three networks, dyads meeting the criteria were scored as 1 and all other dyads were scored as 0. Using multiple regression quadratic assignment procedures (MRQAP), with the proximity bond network as the dependent network, we examined whether these proximity bonds are predicted by other indices of social bonding (visual attention given or received, grooming given, received or mutual, joint travelling, feeding and resting, see Table 2 for definitions of these indices of social bonding). In all these models, we controlled for sex, age, kinship and reproductive similarity. Our results showed that the duration of resting (r^2^ = 0.292, β = 0.220, *p* = 0.007), travelling, (r^2^ = 0.292, β = 0.253, *p* = 0.035), mutual grooming (r^2^ = 0.292, β = 0.169, *p* = 0.049), received grooming (r^2^ = 0.292, β = 0.206, *p* = 0.009) and visual attention given (r^2^ = 0.292, β = 0.276, *p* = 0.031) predicted preferred, reciprocated close proximity bonds. Preferred, non-reciprocated close proximity bonds were predicted by a higher rate of resting in close proximity (r^2^ = 0.188, β = 0.403, *p* = 0.001). The non-preferred close proximity bonds were negatively predicted by higher rates of resting (r^2^ = 0.395, β = −0.530, *p* = 0.001) and travel (r^2^ = 0.395, β = −0.329, *p* = 0.001). These results show that meaningful comparisons across indices of social bondedness can be made using our measure of duration of close proximity per hour spent in the same party.

### Proximity networks and gesture modality

**Figure 1:**
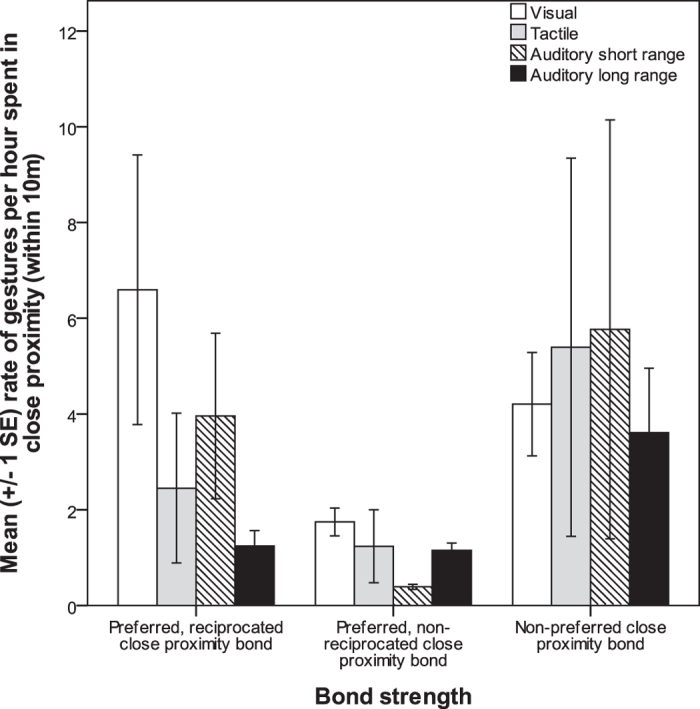
Mean rate of gesture production across modalities, per hour dyad spent in close proximity (within 10 m) for three different types of proximity bond. (1) preferred, reciprocated close proximity bond, where both A to B and B to A dyads had values of close proximity equal or above mean plus half SD (30.3 minutes duration per hour spent in same party); (2) preferred, non-reciprocated close proximity bond, where A to B but not B to A dyads had values of close proximity equal or above mean plus half SD (30.3 minutes duration per hour spent in same party); (3) non-preferred close proximity bond, where A to B dyads had values of close proximity equal or below the mean minus half SD (16.23 minutes duration per hour spent in same party). For the purposes of plotting only, zero values were excluded. The MRQAP regression model included these zero values.

Third, we examined the association between the close proximity bond networks and the rates of different modalities of gestural communication (Fig. 1). A higher rate of visual gestures was significantly associated with the presence of preferred, reciprocated close proximity bonds (r^2^ = 0.173, β = 0.446, *p* = 0.001). In contrast, chimpanzees that were less likely to have preferred, reciprocated close proximity bonds had significantly higher rates of auditory long-range gestures (r^2^ = 0.173, β = −0.267, *p* = 0.001) and tactile gestures (r^2^ = 0.173, β = −0.119, *p* = 0.023). The presence of preferred, non-reciprocated close proximity bonds was not predicted by modality of gestures. Finally, the presence of non-preferred, close proximity bonds was positively predicted by auditory long-range (r^2^ = 0.079, β = 0.191, *p* = 0.039) and tactile gestures (r^2^ = 0.079, β = 0.164, *p* = 0.049) and negatively predicted by visual gestures (r^2^ = 0.079, β = −0.213, *p* = 0.016).

### Proximity centrality and modality

Fourth, we calculated the normalised degree centrality for each individual chimpanzee^61^, i.e. the average value of the strong proximity bond network matrix, where dyads of individuals who had values of proximity association equal or above the mean plus half SD, were scored as 1 (‘strong bonds’). All networks were directed and therefore in-degree and out-degree were calculated separately. Out-degree refers to behaviours directed by the focal chimpanzee to conspecifics, whilst in-degree refers to behaviours directed by conspecifics towards the focal chimpanzee. The proximity network was directed because proximity bonds were not reciprocated in this analysis and therefore out-degree was used in all models.

In these analyses, we used node-level regressions to examine the predictors of proximity out-degree by out- and in-degree of gesture modality. We controlled for the duration of time spent in proximity to oestrus females, time spent in proximity to kin, and the age and sex of the focal chimpanzee. Chimpanzees with a high close proximity out-degree had a high out-degree of auditory long-range gestures (r^2^ = 1, β = 16.547, *p* = 0.011), auditory short-range gestures (r^2^ = 1, β = 2.167, *p* = 0.015) and tactile gestures (r^2^ = 1, β = 8.190, *p* = 0.026). Chimpanzees with high proximity out-degree had a low in-degree of auditory long-range gestures (r^2^ = 1, β = −50.181, *p* = 0.009), auditory short-range gestures (r^2^ = 1, β = −13.891, *p* = 0.010) and tactile gestures (r^2^ = 1, β = −79.099, *p* = 0.009). Chimpanzees with a high proximity out-degree also had a high in-degree (r^2^ = 1, β = 97.751, *p* = 0.009) and low out-degree (r^2^ = 1, β = −39.579, *p* = 0.009) of visual gestures.

## Discussion

In this study, we examined gestural communication where the intended target of communication was within 10 m of the signaller. The gestural communication was examined across pairs of chimpanzees who were in close physical proximity (within 10 m) but varied in the strength of social bonds, as indicated by the duration of time spent in close proximity, visually attending, grooming, traveling and resting. Whilst previous research has shown that primates use a wide variety of gestures in a flexible and intentional way by adjusting the modality of gestures in response to the recipient’s perception^5^,^29^,^80^,^81^,^82^,^83^,^84^,^85^,^86^,^87^,^88^, here we provide the first evidence that this flexibility extends to how gestures of different modalities and intensities are used with different types of social partners.

The pairs of chimpanzees that had a higher rate of visual gestures were more likely to have strong proximity bonds, spending larger amounts of time in close proximity, per hour spent in the same party. The presence of preferred social partners is associated with a lower cardiac arousal response^18^, suggesting that visual gestures are likewise associated with lower levels of arousal. The use of low-arousal visual gestures with strong proximity partners may provide a way for the signaller to effectively convey a rich array of emotional states to the recipient and reduce the inherent stress and risk of aggression involved in being in close proximity. By using visual gestures which are not arousing even in close proximity, these gestures may promote social bonding by coordinating behavior and increasing the predictability of behavior between pairs of regular interaction partners^89^. Visual gestures occurred in both affiliative (e.g. initiating mutual grooming) and antagonistic contexts (e.g. threat to dominate). However, both affiliative and antagonistic contexts can be arousing and elicit positive and negative valence emotions in the recipients. The fact that both contexts were associated with low intensity gestures in pairs of chimpanzees who spent longer time periods in close proximity suggests that social bonding includes managing both competition and affiliation in a non-arousing manner.

However, as compared to high arousal auditory and tactile gestures, visual gestures contain less specific emotional information and require rich contextual information to interpret^54^. Thus, the use of these gestures may be more efficient when used with a small number of regular interaction partners who are more likely to respond to these gestures appropriately, restricting their usefulness in maintaining a larger proximity network of weaker bonds. In contrast, pairs of chimpanzees that spent less time in close proximity had higher rates of auditory long-range and tactile gestures. Previous studies showed that the presence of non-preferred social partners is associated with higher cardiac arousal^18^,^90^,^91^ and therefore these gestures may be underpinned by higher levels of arousal. These gestures may be more effective when communicating with weaker proximity bonds and a greater number of social partners. The use of more intense, higher arousal gestures in this context may reduce the uncertainty when interacting with conspecifics encountered less regularly, a particular issue in fission-fusion social systems. These gestures are less ambiguous in the emotion contained in the signal, as compared to visual gestures, and promote rapid and appropriate responses from the recipients. In particular, long-range auditory gestures such as drumming are an efficient way to communicate over longer distances with many recipients. However, the very features that make this type of communication effective over long distances (e.g. high amplitude) make it arousing to recipients over shorter distances and thus long-range auditory gestures tend to be used where the interests of the signaller and recipient do not overlap (e.g. in aggression contexts by dominant males)^5^,^29^,^92^,^93^. In contrast, tactile gestures were associated with affiliative contexts such as greeting and grooming and thus can also be effective in reestablishing the social relationship with infrequent social interaction partners and reducing uncertainty after a period of absence. The nature of tactile gestures may enable chimpanzees to maintain larger social networks of weak proximity bonds because information about nature of the social relationship and the past history of interactions is not required to infer the emotion from these gestures. However, over longer periods, tactile gestures can cause overstimulation and stress^94^ and thus for regular interaction partners, visual gestures may allow for proximity to be maintained over longer periods in a non-arousing manner.

It could be argued that ecological factors such as food availability or degree of predation risk affected gestural communication indirectly by influencing socio-spatial organisation (e.g. party size and composition, spread, spatial geometry and proximity). For instance, research has shown that in areas where the risk of mortality or injury is high, vulnerable individuals may stay in closer proximity to a dominant ‘protector’ male, party spread may be reduced and proximity between individuals increased^95^,^96^,^97^,^98^,^99^,^100^. Thus socio-environmental conditions can influence the number of recipients who are visually perceptive and in close proximity, which may be reflected in the type and frequency of gestural communication used by a signaller. In this case, when the proximity between the signaller and the recipient is low, the absence of recipients who are proximal and visually perceptive may be associated with a reduction in visual and tactile gestures and a corresponding increase in auditory gestures. In contrast, when the proximity between signaller and recipient is high, there may be an increased frequency of visual and tactile gestural communication. Thus with increasing party size of close proximity partners, the probability of visual and tactile communication would increase and the probability of auditory communication would decline regardless of the strength of bonds between pairs of chimpanzees. However, chimpanzees used communication modalities that were related to the strength of social bonds with the partners, using lower intensity visual gestures with strongly bonded individuals and higher intensity gestures (tactile, auditory) with weakly bonded individuals, regardless of proximity (signallers and recipients were within 10 m of each other in all instances of communication). The fact that an increasing size of the close proximity network is correlated with higher rates of high intensity auditory and tactile gestures and lower rates of low intensity visual gestures provides further evidence that the modality of gestures is related to social bonding in proximity networks of these chimpanzees.

Chimpanzees used long-distance auditory communication such as drumming in relation to the status of the social bond with the partner^92^,^93^,^101^. One interpretation could be that chimpanzees did not communicate to influence the recipient in the immediate audience, but simply coordinated behaviour of the intended recipients in distant parties who were out of sight^102^,^103^,^104^. Drumming can be perceived by several individuals simultaneously and can carry over longer distance which would be beneficial when coordinating movement and facilitating reunions between spatially separated social partners, who may offer support to each other during social conflict^105^,^106^. One piece of evidence to support this claim is that drumming is not visually directed towards members of the same party. The visual attention of the individual towards a social partner indicates the signaller’s interest in behavioural synchrony, which demands that at least one dyad member adjusts their behaviour to match that of their partner. In our study, the pairs of chimpanzees that were more likely to have strong proximity bonds (who spend larger amounts of time in close proximity, per hour spent in the same party) were also more likely to have a longer duration of visual attention directed at the dyad partner, suggesting that visual attention plays an important part in social coordination of wild chimpanzees^29^. When emitted in combination with communication, visual attention serves an important role as a cue enabling the signaller to improve the recipient’s recognition of emotion contained in the signal and hence improve behavioural synchrony^107^,^108^,^109^. For instance, in human communication, simultaneous presentation of emotion in the gesture and direct gaze at the recipient is associated with activation of specific behavioural and brain processes responsible for enhanced emotion pereption^110^,^111^,^112^. However, when emitting high intensity signals, directing visual attention at recipients is not necessary to ensure efficient behavioural coordination with the recipient, because increased arousal is associated with increased specificity of emotion to the dominant signal type and hence the type of emotion contained in the signal is apparent^52^,^66^. Drumming is by far the most intense auditory behaviour of the chimpanzees^105^. Previous studies showed that primates can infer the goal of signalling from knowledge of the past relationships alone when visual attention cues accompanying communication are missing^53^. The absence of visual attention accompanying drumming can therefore be seen as evidence that the presence or absence of directing visual attention at the recipient is interdependent with intensity of arousal in achieving chimpanzee coordination goals^30^,^113^.

Moreover, if chimpanzees used drumming to coordinate behaviour with distal parties rather than coordinate behaviour with social partners in the immediate audience, they should use long-distance auditory communication most frequently in absence of preferred social partners^105^. Contrary to this prediction, previous findings from this population of chimpanzees showed that presence or absence of preferred social partners did not influence the frequency of drumming behaviour^105^. Further, our study showed that the frequency of long-distance auditory gestures is influenced by the presence of non-preferred social partners. This relationship is also valid when considering drumming separately from visually directed auditory gestures^101^. The fact that presence of non-preferred social partners and long-distance auditory gesture networks were correlated shows not only that these gestures were used to communicate with weak social bonds but also that individuals maintain these weak social bonds over time, since the repeated proximity scans picked up the same pattern of weak social bonds as the repeated instances of long-distance auditory gestures. These results strongly suggest that the modality of communication in the current study was shaped by the strength of social bonds between chimpanzees rather than coordination of movement with spatially distant individuals^105^,^114^.

To conclude, the production of low and high arousal gestures in chimpanzees is not simply a readout of the emotional state of the signaller, but instead is related to interacting with different social partners. Further, different modalities of gestures are used across both affiliative and agonistic contexts. Chimpanzees thus appear to have a high degree of flexibility in the production of different types of gestures, including the use of high-intensity gestures and in situations of conflict. This flexibility in the production of gestural communication appears to play a key role in meeting the time and cognitive challenges of managing social relationships in chimpanzees. Given the strong association between individual variation in the strength of social bonds and fitness outcomes^1^, increasing flexibility in the use of different types of communication may have played an important role in social evolution in both primates and hominins. Further studies that account for the role that other communicative channels play in emotion perception, for example the use of vocalizations or facial expressions in decoding emotions contained in low intensity gestures^115^, will help to elucidate the fundamental role that gestural communication plays in regulating social dynamics.

## Methods

The study was approved by the University of Stirling Ethics Committee. The data collection and methods for this study were approved by the Budongo Conservation Field Station research committee. The research was non-invasive and all methods were performed in accordance with the Association for the Study of Animal Behaviour guidelines. We observed twelve East African chimpanzee (*Pan troglodytes schweinfurthii*): six adult males and six adult females at the Budongo Conservation Field Station in Uganda in September 2006, between April and July 2007 and March and June 2008. Full details of the study site, subjects, data collection, video analysis and classification of the gestures have been described previously^5^,^6^,^29^,^54^,^116^, so only brief details are given here. In this study we used quantitative focal animal follows and chose focal subjects systematically, recording focal subject’s behaviour during a standardised observation period of 18 minutes. As far as possible, focal subjects were sampled equally at different times of the day, aiming to sample each focal individual at least once every week. The consecutive samples of the same focal subject were taken at least 20 minutes apart. The data came from two sources. First, 18 minute focal animal follows consisted of 9 scans at 2 minute intervals of the focal individual (i.e. grooming given/received/mutual, identity of grooming partner, presence of travel, resting, feeding, the identity and visual attention of the nearest neighbour towards the focal subject and visual attention of the focal subject towards the nearest neighbour) and the identity of the individuals present within 10 m of the focal subject and who were more than 10 m away, but who were in the same party. The party was defined as the group of individuals within a spread of around 35 m. Second, the chimpanzee behaviour was continuously videotaped and contained a verbal description of context: the identity of the signaller/recipient, their behaviour prior to and after production of the gesture, goal directedness, proximity and bodily orientation between signaller and the recipient. This was almost always possible with the aid of the field assistant since chimpanzees approach the recipient before signaling^117^ and gesture towards nearest neighbor^29^ from a mean distance of 6.4 meters^29^. However, if the recipient of the gesture or context could not be established, we excluded these gestures from the analyses. In those cases when the gestures were not visually directed (e.g. drumming) we used the identity of the nearest neighbor to determine proximity, bodily orientation and context according with the established methods in vocal behaviour research^118^. All individuals within 10 m were designated as the recipient of the drumming behaviour.

Next, the video footage was viewed on a television and coded. The types of non-verbal behaviours were established qualitatively on the basis of objective judgment of the similarity of morphology (i.e. presence/absence and type of head, trunk, arm movement; posture, social orientation). The nonverbal behaviors were scored as an act of gestural communication if the following criteria, established in previous developmental research, were met^81^,^119^,^120^,^121^,^122^,^123^,^124^,^125^,^126^: (1) the behaviour was an expressive movement of the limbs or head and body posture that was mechanically ineffective (a gesture always elicited a change in recipient’s behaviour by non-mechanical means), (2) behaviour was communicative (i.e. produced a change in the behaviour of recipient on at least 60% of occasions)^127^ and (3) behaviour was goal-directed (intentional)^87^,^119^,^120^,^121^,^122^,^123^,^124^,^125^,^126^,^128^,^129^,^130^,^131^,^132^. The behaviour was intentional when there was: (1) presence of immediate audience (within 10 m), (2) response waiting (the signaller was visually oriented in the direction of the recipient when producing a gesture towards a recipient and continued to be visually oriented after the gesture); (3) the production of a gesture was sensitive to the recipient’s visual attention state (recipient was visually oriented in the direction of the signaller when the gesture was made with possible exception of tactile or auditory gestures); (4) the signaller persisted in gesture production when the recipient failed to respond and stopped gesturing when recipient responded to the gesture. All of these intentionality criteria were evaluated for each gesture type separately, using pooled data across all subjects. Gestures above the threshold of 60% of cases were classified as intentional. It is currently debated whether intentionality criterion should be met by each case of behaviour for communication to be considered intentional^86^,^133^. In all previous studies of gestural communication in great apes, sequences were included in the dataset although sequences were not always formed by communicative persistence or response waiting p. 833^84^, see also^5^,^87^,^134^,^135^. Following these established methods in previous studies, we required that at least one intentionality criterion (criteria 2–4) had to be met for the communication to be considered intentional at the level of 60% of the cases^132^,^136^. This did not include the criterion ‘audience presence’ in this dataset and the recipient was always present within 10 m for this analysis.

Gestures occur singly or in sequences, defined as one or more than one gesture made consecutively by one individual, towards the same recipient, the same goal, within the same context, within a maximum of 30 s interval. For each single gesture or sequence we recorded: identity of the signaller (the individual performing a gesture); identity of the recipient (individual at whom the gesture was most clearly directed, as determined from the orientation of head and body of the signaller during or immediately after performing a gesture, i.e. the signaller had the recipient within its field of view) and the function (see Table 2 for ethogram). Moreover, gestures were classified according to the modality following the ethogram detailed in Table 1. To avoid the same gesture events appearing in multiple modality categories, only gesture events that could exclusively be assigned to one of the four gesture modalities were included in this analysis. Moreover, only those independent gesture events were included where the recipient was within 10 m of the signaller during the gesture production, to control for the ability of the recipient to perceive the gesture. This formed basis for the creation of behavioural measures described in Supplementary Information S1. All behavioural measures in this study were based on duration in terms of proximity, visual attention, grooming, travel, feeding and resting (minutes spent in activity, per hour spent in the same party) or rates (frequency of gestures produced, per hour spent in close proximity–within 10 m). The chimpanzee dyads were categorized according to kinship similarity, sex similarity, reproductive similarity and age proximity following previous studies^69^. The details of this categorization can be found in Supplementary Information S1.

### Social Network Analysis

Proximity and communication networks were created for each behaviour type separately. The matrices consisted of 12 rows and 12 columns, where each row and column denoted a different focal chimpanzee. The values in each cell of the matrix represented the value for that particular behaviour for a specific pair of chimpanzees (e.g. the duration of time spent in close proximity, per hour spent in the same party, between Bwoba and Hawa). All the communication networks used in the analyses were weighted, that is each cell consisted of a continuous value representing the behaviour, rather than a 1 or a 0 indicating the presence or absence of a tie. Moreover, all of the communication networks were directed. For instance, the rate of gestures produced by Bwoba that were directed to Hawa may be different to the rate of gestures by Hawa that were directed to Bwoba. From these network matrices, centrality measures were calculated, using normalized degree centrality^61^. Normalised degree centrality is defined as the average value of each row or column of the network matrix i.e. the average value of that behaviour for each focal chimpanzee. In all instances, the communication networks were directed (i.e. the rate of visual gesture networks) and therefore in-degree and out-degree were calculated separately. Out-degree refers to behaviours directed by the focal chimpanzee to conspecifics, whilst in-degree refers to behaviours directed by conspecifics towards the focal chimpanzee.

In order to provide descriptive data on the normalized mean degree across the behavioral networks (e.g. visual and tactile gestures), we dichotomized and symmetrized the networks^137^. This enables easier interpretation of the normalised mean degrees, which is the mean proportion of all possible ties which are present. In order to dichotomize the network, all values over zero were scored as 1 (present) and all values of zero were classed as absent. For symmetrisation, a tie was scored as present if there was a 1 in either of the two cells corresponding to each pair of individuals (cell *i, j* or cell *j, i*). General standard inferential statistics cannot be used on network data because the observations that make up network data are not independent of each other. Thus, randomisation (or permutation) tests are used, whereby the observed value is compared against a distribution of values generated by a large number of random permutations of the data. The proportion of random permutations in which a value as large (or as small) as the one observed is then calculated, and this provides the *p* value of the test^137^. MRQAP regression (Multiple Regression Quadratic Assignment Procedure) was used to determine the relationships between behavioural networks^137^. MRQAP regression is similar to standard regression because it enables the examination of the effect of a number of predictor variables (e.g. visual communication network, control variables) on an outcome variable (e.g. proximity network). Amongst several different types of MRQAP regression that are available, we used Double Dekker Semi-Partialling MRQAP regression, because this regression is robust against the effects of network autocorrelation and skewness in the data. The number of permutations used in this analysis was 2,000. For the node-level regressions, we used a similar procedure, using 10,000 random permutations to assess the effect of a number of predictor variables (e.g. the out-degree for gestures, sex of focal chimpanzee) on the outcome variable (e.g. proximity out degree).

Finally, to assess autocorrelation between attribute data (e.g. the total duration of observation) and network data (e.g. visual gesture network), the Geary’s C statistic was used. When there is no association between variables, the Geary statistic has a value of 1.0, with values of less than 1.0 indicating a positive association and values over 1.0 indicating a negative association. UCINET 6 for Windows was used to carry all data transformations and analyses^138^.

## Additional Information

**How to cite this article**: Roberts, A. I. and Roberts, S. G. B. Wild chimpanzees modify modality of gestures according to the strength of social bonds and personal network size. *Sci. Rep.* **6**, 33864; doi: 10.1038/srep33864 (2016).

## Supplementary Material

Supplementary Information

## Figures and Tables

**Table 1:** Modality ethogram, accompanying gesture types, signaller–recipient distance, rate and normalized degree (percentage of all connections chimpanzees had with others) across 132 chimpanzee dyads

Definition	Gesture types*	Gesture rate/dyad (overall range)	N degree (%)/dyad (overall range)	Mean ± SD distance between signaller and the recipient
**Visual gestures**				
Gesture perception is possible only by looking at signaller	Arm beckon, Arm flap, Arm raise, Bob, Bow, Crouch, Crouch run, Crouch walk, Dangle, Forceful extend, Hand bend, Jump, Limp extend, Linear sweep, Lower head, Lunge, Present genitals, Present leg, Present mount, Present rump, Present torso, Rock, Roll over, Run stiff, Slap self, Sniff, Stationary stiff, Stiff extend, Stretched extend, Swagger bipedal, Swagger quadrupedal, Tip head, Touch self, Turn back, Turn head, Unilateral swing, Vertical extend, Walk stiff, Wipe	1.56 (0–42.5)	48 (9–100)	3.03 ± 4.15
**Tactile gestures**				
Gesture perception is possible via physical contact	Bite, Embrace, Grab, Hold hands, Kiss, Locomote tandem, Pull another, Push by hand, Push by rump, Rub, Shake limb, Slide, Stand tandem, Stroke by mouth, Tap another, Thrust genitals, Tickle, Touch backhand, Touch innerhand, Touch long	0.34 (0–21.17)	18 (0–64)	1.76 ± 3.46
**Auditory short-range gestures**				
Sounds produced by the gesture can be heard within short distance from the signaller up to 10 meters	Clip by mouth, Smack lip, Tap object	0.4 (0–23.12)	18 (0–36)	0.19 ± 0.74
**Auditory long-range gestures**				
Sounds produced are audible at a distance of more than 10 meters away from the recipient	Beat, Bounce, Drum, Knock, Pound, Shake mobile, Shake stationary, Stamp quadrupedal, Stamp sitting, Sway, Swing	0.4 (0–15)	27 (0–55)	5.47 ± 3.04

^*^Description and video footage of gesture types can be found in Roberts A.I., Roberts S.G.B., Vick S.-J. 2014 The repertoire and intentionality of gestural communication in wild chimpanzees. Animal Cognition 17, 317–336 and Roberts A.I., Vick S.-J., Roberts S.G.B., Buchanan-Smith H.M., Zuberbühler K. 2012 A structure-based repertoire of manual gestures in wild chimpanzees: statistical analyses of a graded communication system. *Evolution and Human Behavior* 33(5), 578–589.

**Table 2:** Categorization of gestures according to function.

Category of gesture	Definition
**Gestures**	
Threat to dominate	Aggressive context, where there is no tangible reason for conflict but the recipient reacts with fear (e.g. screams)
Food sharing	Context where food is in recipient’s possession and in view of the signaller who makes gestures in anticipation of receiving food item.
Other threat	Communication motivated by clear conflict of interest over the resource such as food or behavior such as mating
Travel	Gestures motivated by the signaller’s desire to be followed by the recipient from one location to the next.
Copulation	Gestures produced by a male or a tumescent female in order to initiate copulation
Reassurance	Gestures produced in reaction to recipient’s distress, fright or hurt by the signallers own behaviour or third party threat.
Greeting	Gestures accompanying approaching, being approached or leaving approach with the recipient who is non-threatening or when the recipient or third party distressed, frightened or hurt the signaller.
Mutual groom	Gestures made to initiate simultaneous grooming between signaller and the recipient.
Receive groom	Gestures made to initiate grooming of the signaller by the recipient.
Give groom	Gestures made to initiate grooming of the recipient by the signaller.
Play	Gestures which initiate bouts of wrestling, chasing, tickling in non-agonistic relaxed manner accompanied by play-face.
Pant-hoot	Production of pant hoot call solo or jointly with others when accompanied by production of gestures
**Other bonding behaviors**	
Travel	Focal subject travels within 10 m of non-focal subject
Resting	Focal subject rests within 10 m of the non-focal subject
Feeding	Focal subject feeds within 10 m of the non-focal subject
Mutual grooming	Focal individual simultaneously grooms with non-focal subject
Received grooming	Focal individual receives grooming from non-focal subject
Given grooming	Focal individual grooms non-focal subject
Visual attention given	Focal individual visually monitors non-focal subject who is its nearest neighbour and within 10 m
Visual attention received	Non-focal individual visually monitors focal subject who is its nearest neighbour and within 10 m

**Table 3:** Contextual predictors of rates of gestural communication between focal chimpanzees, per hour spent in close proximity (within 10 m).

Rate of gestural communication	Standardized coefficient	Standard error	*P*
Long-range auditory gestures			
Threat to dominate	0.479	0.594	0.009
Other threat	0.368	0.294	0.001
Travel	0.037	0.276	0.046
Copulation	0.103	0.093	0.005
Reassurance	−0.367	0.530	0.043
Gesture to receive groom	−0.171	0.175	0.018
Pant-hoot	0.866	0.128	0.001
Short-range auditory gestures			
Threat to dominate	0.240	0.423	0.008
Other threat	−0.025	0.190	0.043
Copulation	0.017	0.074	0.046
Reassurance	−0.172	0.372	0.044
Greeting	0.042	0.053	0.007
Gesture to give groom	0.985	0.136	0.001
Play	−0.101	0.036	0.001
Tactile gestures			
Threat to dominate	−0.106	0.290	0.039
Reassurance	0.486	0.298	0.001
Greeting	0.113	0.071	0.001
Gesture to give groom	0.035	0.020	0.024
Play	0.875	0.104	0.001
Visual gestures			
Threat to dominate	0.205	1.011	0.014
Food sharing	0.017	5.125	0.043
Other threat	0.142	0.472	0.008
Travel	0.087	0.485	0.012
Copulation	0.152	0.209	0.008
Greeting	0.201	0.261	0.001
Gesture to mutually groom	0.373	1.367	0.032
Gesture to receive groom	0.138	0.307	0.015
Play	0.079	0.086	0.011
Pant-hoot	0.477	0.181	0.001

Only significant predictors are shown in this table with full models presented in SI, Tables S3–S6.
