# Supplementary material for: Wild chimpanzees modify modality of gestures according to the strength of social bonds and personal network size
Source: Sci Rep. 2016 Sep 21;6:33864. doi: 10.1038/srep33864 (PMC5030607; doi:10.1038/srep33864)
Supplement: Supplementary Information [file srep33864-s1.pdf]

Supplementary Information

Wild chimpanzees modify modality of gestures according to the strength of social bonds and personal network size

Anna Ilona Roberts, Sam George Bradley Roberts

Supplementary Information S1

Dependence of the proximity data collection

In order to ensure that this sampling procedure did not bias our results, we tested similarity in association patterns between scans taken at 2 minutes (scan 1), 4 minutes (scan 2) and 18 minutes (scan 9) of the focal sample, including both sexes. There was no significant difference in the number of times focal and non-focal subjects were in close proximity at scan 1 (Median = 2, IQ range = 0 - 5) and scan 2 (Median = 2, IQ range = 1 – 5, Wilcoxon signed-ranks test, $T = 411.50$, $n = 132$, $p = 0.435$). However, there was a significant difference in the number of times focal and non-focal subjects were in close proximity at scan 1 and scan 9 (Median = 2, IQ range = 1 – 4; Wilcoxon signed-ranks test, $T = 2656.50$, $n = 132$, $p = 0.011$). Similarly, there was no significant difference in the number of times focal and non-focal subjects were in the same party at scan 1 (median = 5, IQ range: 3 - 10) and scan 2 (median = 5, IQ range: 3 - 10; Wilcoxon signed-ranks test, $T = 218.50$, $n = 132$, $p = 0.571$). However, there was a significant difference in the number of times focal and non-focal subjects were in the same party at scan 1 and scan 9 (median = 5, IQ range: 2 - 10; Wilcoxon signed-ranks test, $T = 1460$, $n = 132$, $p = 0.010$). Thus, the adjacent scans were similar for 10m associations and party level associations and were treated as continuous. However, the first and final sample scans differed for 10m associations and party level associations and therefore we established that these scans were independent, as well as the samples preceding and succeeding the focal follow.

The behavioural measures were then calculated in the following manner:

The dyadic association measure

The dyadic association measure (DA) is the duration of time focal subject A spent in close proximity (within 10m) to non-focal subject B per hour spent in the same party, or:

$DA_{AB} = [(P10_{AB}*2)* 60)] /PSP_{AB}*2$

where $P10_{AB}$ = the number of times A was in close proximity (within 10m) to B

PSP_{AB} = the number of times A was in the same party as B

2 = duration of instantaneous subsample interval in minutes

60 = the number of minutes in an hour

Note that the multiplication by 60 enabled meaningful comparisons between indices (see below). Moreover, this controls for the number of times proximity overlapped, resulting in an index that is independent.

The dyadic association measure between kin

The dyadic association measure between kin (DAK) is the duration of time focal subject A spent in close proximity (within 10m) to non-focal subject B, who is related to A, per hour spent in the same party, or:

$DAK_{AB} = [(P10_{AB}*2)* 60)] /PSP_{AB}*2$

where $P10_{AB}$ = the number of times A was in close proximity (within 10m) to B, who is related to A

PSP_{AB} = the number of times A was in the same party as B who is the kin

2 = duration of instantaneous subsample interval in minutes

60 = the number of minutes in an hour

The dyadic association measure of the oestrous female

The dyadic association measure of the oestrous female (DAR) is the duration of time focal subject A who is a female exhibiting sexual swelling in the final phase of tumescence spent in close proximity (within 10m) to non-focal subject B, per hour spent in the same party, or:

$DAR_{AB} = [(P10_{AB}*2)* 60)] /PSP_{AB}*2$

where $P10_{AB}$ = the number of times A (who is oestrous female) was in close proximity (within 10m) to B

PSP_{AB} = the number of times A was in the same party as B

2 = duration of instantaneous subsample interval in minutes

60 = the number of minutes in an hour

<u>The dyadic communication measure</u>

The dyadic communication measure (CA) is rate at which focal subject A communicated to non-focal subject B when B was in close proximity (within 10m) to focal subject A, per hour spent within 10 m of the non-focal subject B, or:

$GA_{AB} = (C_{AB}* 60) / P10_{AB} *2$

where C_{AB} = the number of times A communicated with B when in close proximity (within 10m) to B

$P10_{AB}$ = the number of times A was in close proximity (within 10m) to B

2 = duration of instantaneous subsample interval in minutes

60 = the number of minutes in an hour

<u>The dyadic grooming measure</u>

The dyadic grooming measure (GA) is the duration of time focal subject A spent grooming with non-focal subject B when B was in close proximity (within 10m) to focal subject A, per hour spent within 10 m of the non-focal subject B, or:

$GA_{AB} = [(GR_{AB}*2)*60] / P10_{AB} *2$

where GR_{AB} = the number of times A grooming with B when in close proximity (within 10m) to B

$P10_{AB}$ = the number of times A was in close proximity (within 10m) to B

2 = duration of instantaneous subsample interval in minutes

60 = the number of minutes in an hour

<u>The dyadic activity measure (calculated in the same way for feeding, resting and travel)</u>

The dyadic activity measure (AA) is the duration of time focal subject A spent in a particular activity when B was in close proximity (within 10m) to focal subject A, per hour spent in the same party as B, or:

$AA_{AB} = [(AR_{AB}*2)*60] / PSP_{AB} *2$

where AR_{AB} = the number of times A performed activity with B when in close proximity (within 10m) to B

PSP_{AB} = the number of times A was in the same party as B

2 = duration of instantaneous subsample interval in minutes

60 = the number of minutes in an hour

<u>The dyadic visual attention given measure</u>

The dyadic visual attention given measure (VAG) is the duration of time focal subject A spent visually oriented towards nearest neighbour B per hour spent within 10 meters to B, or:

$VAG_{AB} = [(VRG_{AB}*2)*60] / P10_{AB} *2$

where VRG_{AB} = the number of times A was visually oriented towards nearest neighbour B when in close proximity (within 10m) to B

$P10_{AB}$ = the number of times A was in close proximity (within 10m) to nearest neighbour B

2 = duration of instantaneous subsample interval in minutes

60 = the number of minutes in an hour

<u>The dyadic visual attention received measure</u>

The dyadic visual attention received measure (VAR) is the duration of time focal subject A received visual orientation from the nearest neighbour B per hour spent within 10 meters to B, or:

$VAR_{AB} = [(VRR_{AB}*2)*60] / P10_{AB} *2$

where VRR_{AB} = the number of times A received visual orientation from the nearest neighbour when in close proximity (within 10m) to B

$P10_{AB}$ = the number of times A was in close proximity (within 10m) to nearest neighbour B

2 = duration of instantaneous subsample interval in minutes

60 = the number of minutes in an hour

Attribute measures

Previously known maternal kinship was used to classify pairs (dyads) of chimpanzees as maternal kin or non-kin [1]. Wild chimpanzees attain physical and social maturity between ages 15 – 16 years old [2]. These ages of the chimpanzees in the Sonso community were known. The chimpanzee dyads were classified as belonging to the same (5 years or less age difference) or a different (above 5 years age difference) age class[3]. Moreover, the reproductive similarity was scored assessing reproductive status of the female on the basis of the size of sexual swelling (the enlarged area of the perineal skin varying in size over the course of the menstrual cycle). The reproductive status of the female was deemed as oestrous if during the observation period the female exhibited maximum tumescence and was observed mating with the males. All focal males were reproductively active (physically mature and observed to mate with females). The full details of the categorization of attribute data are provided in Supplementary Table S2.

Inter-observer reliability

An experienced field assistant sampled behaviour and association patterns but was unaware of the aims of the study. The field assistant undergoes an inter-observer reliability test with

other field assistants from the project annually, the sufficient interval to maintain the consistency of scoring of the group composition, proximity and activity across field assistants, with results consistently above 0.85 Spearman's rank correlation coefficient, r_S. The filming of video footage and context commentary was carried out by AR and therefore the social data and gestural data were collected independently of each other. The coding was validated by the second coder who scored a random sample of 10.42% of the gesture sequences, coding the function and modality of gesture, assigning them correctly to each of the categories. Cohen's Kappa coefficient showed that reliability was excellent for function (K = 0.70) and modality of gesturing (K = 0.946)[4].

Supplementary Table S2 Categorisation of attributes

Category of attribute	Subcategory of attribute	Description of subcategory
Kinship similarity	Maternal kin	mother-son<br>son-mother
	Maternal non-kin	not a mother - son or son-mother dyad
Sex similarity	Same sex	male-male<br>female-female
	Different sex	male-female<br>female-male
Reproductive similarity	Same reproductive state	male-male<br>male-oestrous female<br>oestrous female - male<br>unoestrous female – unoestrous female<br>oestrous female- oestrous female
	Different reproductive state	unoestrous female-oestrous female<br>unoestrous female-male
Age proximity	Same age	no more than 5 years age difference between individuals

	category	in the dyad
	Different age category	more than 5 years age difference between individuals in the dyad
Sex	Male<br>Female	
Age	Young adult	16 years old or younger
	Mature adult	More than 16 years of age

Supplementary Table S3 MRQAP regression model showing function predictors of long-range auditory gestures between N = 12 focal chimpanzees, 132 dyadic relationships of the chimpanzees. Significant P values are indicated in bold. Description of gesture functions is given in Table 2.

Attribute category/ rate or duration of behaviour per hour spent in close proximity	r^2 = 0.868		
	Standardized coefficient	Standard error	*P*
Reproductive similarity	-0.025	0.136	0.266
Age similarity	-0.065	0.189	0.054
Kinship similarity	0.008	0.320	0.359
Sex similarity	0.006	0.154	0.441
Threat to dominate	0.479	0.594	**0.009**
Food sharing	0.016	3.201	0.137
Other threat	0.368	0.294	**0.001**
Travel	0.037	0.276	**0.046**
Copulation	0.103	0.093	**0.005**
Reassurance	-0.367	0.530	**0.043**
Greeting	-0.010	0.081	0.306
Gesture to mutually groom	0.050	0.677	0.370
Gesture to receive groom	-0.171	0.175	**0.018**

Gesture to give groom	0.013	0.038	0.297
Play	0.032	0.043	0.057
Pant-hoot	0.866	0.128	**0.001**

Supplementary Table S4 MRQAP regression model showing context predictors of short-range auditory gestures between N = 12 focal chimpanzees, 132 dyadic relationships of the chimpanzees. Significant P values are indicated in bold. Description of gesture functions is given in Table 2.

Attribute category/ rate or duration of behaviour per hour spent in close proximity	$r^2 = 0.971$		
	Standardized coefficient	Standard error	*P*
Reproductive similarity	-0.027	0.109	0.094
Age similarity	-0.005	0.127	0.379
Kinship similarity	0.003	0.224	0.520
Sex similarity	0.014	0.100	0.246
Threat to dominate	0.240	0.423	**0.008**
Food sharing	0.004	2.948	0.294
Other threat	-0.025	0.190	**0.043**
Travel	-0.004	0.241	0.183
Copulation	0.017	0.074	**0.046**
Reassurance	-0.172	0.372	**0.044**
Greeting	0.042	0.053	**0.007**
Gesture to mutually groom	-0.015	0.461	0.409

Gesture to receive groom	0.016	0.105	0.226
Gesture to give groom	0.985	0.136	**0.001**
Play	-0.101	0.036	**0.001**
Pant-hoot	0.004	0.035	0.373

Supplementary Table S5 MRQAP regression model showing context predictors of tactile gestures between N = 12 focal chimpanzees, 132 dyadic relationships of the chimpanzees. Significant P values are indicated in bold. Description of gesture functions is given in Table 2.

Attribute category/ rate or duration of behaviour per hour spent in close proximity	$r^2 = 0.977$		
	Standardized coefficient	Standard error	*P*
Reproductive similarity	0.019	0.065	0.078
Age similarity	-0.027	0.090	**0.047**
Kinship similarity	0.002	0.155	0.393
Sex similarity	0.018	0.077	0.133
Threat to dominate	-0.106	0.290	**0.039**
Food sharing	0.001	1.536	0.340
Other threat	0.000	0.100	0.415
Travel	0.009	0.119	0.090
Copulation	0.007	0.047	0.154
Reassurance	0.486	0.298	**0.001**
Greeting	0.113	0.071	**0.001**
Gesture to mutually groom	-0.009	0.375	0.434

Gesture to receive groom	0.021	0.078	0.141
Gesture to give groom	0.035	0.020	**0.024**
Play	0.875	0.104	**0.001**
Pant-hoot	0.007	0.023	0.157

Supplementary Table S6 MRQAP regression model showing context predictors of visual gestures between N = 12 focal chimpanzees, 132 dyadic relationships of the chimpanzees. Significant P values are indicated in bold. Description of gesture functions is given in Table 2.

Attribute category/ rate or duration of behaviour per hour spent in close proximity	r^2 = 0.939		
	Standardized coefficient	Standard error	*P*
Reproductive similarity	-0.034	0.237	0.059
Age similarity	-0.042	0.329	**0.046**
Kinship similarity	-0.006	0.553	0.367
Sex similarity	0.002	0.277	0.470
Threat to dominate	0.205	1.011	**0.014**
Food sharing	0.017	5.125	**0.043**
Other threat	0.142	0.472	**0.008**
Travel	0.087	0.485	**0.012**
Copulation	0.152	0.209	**0.008**
Reassurance	0.073	1.068	0.248
Greeting	0.201	0.261	**0.001**

Gesture to mutually groom	0.373	1.367	**0.032**
Gesture to receive groom	0.138	0.307	**0.015**
Gesture to give groom	-0.014	0.072	0.188
Play	0.079	0.086	**0.011**
Pant-hoot	0.477	0.181	**0.001**

Supplementary Table S7. MRQAP regression model showing predictors of proximity between N = 12 focal chimpanzees, 132 dyadic relationships of the chimpanzees. Significant P values are indicated in bold. Dyads of individuals who had values of proximity association equal or greater than the mean plus half SD, were scored as 1 if the proximity was reciprocated (i.e. both A to B and B to A displayed values of proximity association equal or greater than the mean plus half SD: 'preferred, reciprocated close proximity ties') whereas other dyads were scored as 0. Description of behavioral categories is given in Table 2.

Attribute category/ duration of behaviour per hour spent in close proximity	r^2 =0.292		
	Standardized coefficient	Standard error	*P*
Kinship similarity	-0.091	0.202	0.214
Reproductive similarity	-0.192	0.094	0.062
Age similarity	0.053	0.114	0.330
Sex similarity	0.054	0.090	0.303
Visual attention given	0.276	0.004	**0.031**
Visual attention received	-0.185	0.004	0.083
Grooming mutual	0.169	0.015	**0.049**
Grooming received	0.206	0.014	**0.009**

Grooming given	0.018	0.014	0.369
Travel	0.253	0.016	**0.035**
Resting	0.220	0.009	**0.007**
Feeding	-0.141	0.002	0.143

Supplementary Table S8. MRQAP regression model showing predictors of proximity between N = 12 focal chimpanzees, 132 dyadic relationships of the chimpanzees. Significant P values are indicated in bold. Dyads of individuals who had values of proximity association equal or greater than the mean plus half SD, were scored as 1 if the proximity was non-reciprocated (i.e. only A to B but not B to A displayed values of proximity association equal or greater than the mean plus half SD – 'preferred, non-reciprocated close proximity ties') other dyads were scored as 0. Description of behavioral categories is given in Table 2.

Attribute category/ duration of behaviour per hour spent in close proximity	r^2 =0.188		
	Standardized coefficient	Standard error	*P*
Kinship similarity	0.099	0.178	0.121
Reproductive similarity	0.073	0.061	0.151
Age similarity	-0.054	0.093	0.288
Sex similarity	0.000	0.077	0.487
Visual attention given	-0.106	0.004	0.209
Visual attention received	0.052	0.005	0.332
Grooming mutual	-0.048	0.015	0.309
Grooming received	0.006	0.016	0.435

Grooming given	-0.011	0.017	0.480
Travel	0.021	0.017	0.424
Resting	0.403	0.011	**0.001**
Feeding	-0.052	0.002	0.372

Supplementary Table S9. MRQAP regression model showing predictors of proximity between N = 12 focal chimpanzees, 132 dyadic relationships of the chimpanzees. Significant P values are indicated in bold. Dyads of individuals who had values of proximity association equal or lower than the mean plus half SD, were scored as 1 ('non-preferred close proximity ties', i.e. A to B displayed values of proximity association equal or lower than the mean plus half SD) whereas other dyads were scored as 0. Description of behavioral categories is given in Table 2.

Attribute category/ duration of behaviour per hour spent in close proximity	r^2 =0.395		
	Standardized coefficient	Standard error	*P*
Kinship similarity	-0.043	0.190	0.285
Reproductive similarity	-0.039	0.081	0.308
Age similarity	-0.157	0.102	**0.031**
Sex similarity	0.097	0.078	0.098
Visual attention given	0.021	0.005	0.414
Visual attention received	0.082	0.005	0.234
Grooming mutual	-0.063	0.016	0.204
Grooming received	-0.102	0.018	0.084
Grooming given	-0.042	0.017	0.311

Travel	-0.329	0.018	**0.001**
Resting	-0.530	0.012	**0.001**
Feeding	0.095	0.002	0.223

Supplementary Table S10. MRQAP regression model showing predictors of proximity between N = 12 focal chimpanzees, 132 dyadic relationships of the chimpanzees. Significant P values are indicated in bold. Dyads of individuals who had values of proximity association equal or greater than the mean plus half SD, were scored as 1 if the proximity was reciprocated (i.e. both A to B and B to A displayed values of proximity association equal or greater than the mean plus half SD: 'preferred, reciprocated close proximity ties') whereas other dyads were scored as 0. Description of gesture modalities is given in Table 1.

Attribute category/ rate of gesture sequence per hour spent in close proximity	r^2 =0.173		
	Standardized coefficient	Standard error	*P*
Reproductive similarity	-0.154	0.100	0.118
Age similarity	0.083	0.111	0.250
Kinship similarity	-0.072	0.205	0.324
Sex similarity	0.075	0.092	0.281
Auditory long-range	-0.267	0.022	**0.001**
Auditory short-range	0.063	0.016	0.221
Tactile	-0.119	0.019	**0.023**
Visual	0.446	0.011	**0.001**

Supplementary Table S11. MRQAP regression model showing predictors of proximity between N = 12 focal chimpanzees, 132 dyadic relationships of the chimpanzees. Significant P values are indicated in bold. Dyads of individuals who had values of proximity association equal or greater than the mean plus half SD, were scored as 1 if the proximity was non-reciprocated (i.e. only A to B but not B to A displayed values of proximity association equal or greater than the mean plus half SD – 'preferred, non-reciprocated close proximity ties') other dyads were scored as 0. Description of modalities is given in Table 1.

Attribute category/ rate of gesture sequence per hour spent in close proximity	r^2 =0.026		
	Standardized coefficient	Standard error	*P*
Age similarity	-0.044	0.087	0.311
Sex similarity	-0.017	0.076	0.431
Kinship similarity	0.073	0.155	0.198
Reproductive similarity	0.077	0.061	0.130
Auditory long-range	-0.043	0.025	0.385
Auditory short-range	-0.055	0.017	0.343
Tactile	-0.020	0.020	0.608
Visual	-0.046	0.010	0.375

Supplementary Table S12. MRQAP regression model showing predictors of proximity between N = 12 focal chimpanzees, 132 dyadic relationships of the chimpanzees. Significant P values are indicated in bold. Dyads of individuals who had values of proximity association equal or lower than the mean plus half SD, were scored as 1 ('non-preferred close proximity ties', i.e. A to B displayed values of proximity association equal or lower than the mean plus half SD) whereas other dyads were scored as 0. Description of gesture modalities is given in Table 1.

Attribute category/ rate of gesture sequence per hour spent in close proximity	r^2 =0.079		
	Standardized coefficient	Standard error	*P*
Age similarity	-0.155	0.120	0.058
Sex similarity	0.080	0.098	0.208
Kinship similarity	-0.082	0.212	0.194
Reproductive similarity	-0.045	0.098	0.339
Auditory long-range	0.191	0.030	**0.039**
Auditory short-range	-0.062	0.020	0.312
Tactile	0.164	0.024	**0.049**
Visual	-0.213	0.013	**0.016**

Supplementary Table S13. Node level regression predicting proximity out degree between N = 12 focal chimpanzees. Significant *P* values are given in bold (r^2 =1). Normalised degree centrality is the average value of each row or column of the network matrix i.e. the average value of that behaviour for each focal chimpanzee. The networks are directed, therefore in degree and out degree are calculated separately. Out degree refers to behaviours directed by the focal chimpanzee to conspecifics, whilst in degree refers to behaviours directed by conspecifics towards the focal chimpanzee. Description of gesture modalities is given in Table 1.

Variable	Standardized coefficient	*P*
Sex/ age	4.804	**0.007**
Kinship	-0.937	0.145
Reproductive state of female	-0.181	0.395
Auditory long-range out-degree	16.547	**0.011**
Auditory long-range in-degree	-50.181	**0.009**
Auditory short-range out-degree	2.167	**0.015**
Auditory short-range in-degree	-13.891	**0.010**
Tactile out-degree	8.190	**0.026**
Tactile in-degree	-79.099	**0.009**
Visual out-degree	-39.579	**0.009**
Visual in-degree	97.751	**0.009**

References

1 Reynolds, V. *The chimpanzees of the Budongo Forest: Ecology, behaviour, and conservation*. (Oxford University Press, 2005).

2 Goodall, J. *The Chimpanzees of Gombe: Patterns of Behaviour*. (Harward University Press, 1986).

3 Mitani, J. C., Watts, D. P., Pepper, J. W. & Merriwether, D. A. Demographic and social constraints on male chimpanzee behaviour. *Animal Behaviour* **64**, 727-737 (2002).

4 Bakeman, R. & Gottman, J. M. *Observing Interaction: An Introduction to Sequential Analysis*. (Cambridge University Press, 1997).
